# Light Metabolically Reprograms CD8^+^ T Cells to Potentiate STING‐Driven Tumor Eradication and Prevent Metastasis

**DOI:** 10.1002/advs.202515121

**Published:** 2025-10-23

**Authors:** Asmita Banstola, Shilin Gao, Zhengkung Zhang, Yan Dong, Prabhat Upadhyay, Quanwei Zhang, Yongli Li, Zuan‐Tao Lin, Zhilong Wang, Mei X. Wu

**Affiliations:** ^1^ Wellman Center for Photomedicine Massachusetts General Hospital Department of Dermatology Harvard Medical School 50 Blossom Street Boston MA 02114 USA

**Keywords:** cGAS–STING pathway, photo‐biomodulation, resident memory T cells, tumor microenvironment

## Abstract

Immunotherapy remains ineffective in many solid tumors due to poor T‐cell infiltration and a metabolically suppressive tumor microenvironment. A dual strategy combining low‐level light (LLL) therapy with a nanoscale stimulator of interferon genes (STING) agonist formulation (nanoSTING@Mn) is presented to enhance immune activation and metabolic fitness for durable tumor immunity against T‐cell lymphoma (EL4) model. NanoSTING@Mn, composed of ADU‐S100 complexed with Mn^2^⁺ and encapsulated in biomimetic liposomes, potently activates the cGAS–STING pathway, induces a type I interferon response, and promotes lymphocyte infiltration. These monocytes polarize into M1 macrophages, suppressing regulatory T cells. Simultaneously, LLL photo‐biomodulation reprograms mitochondrial metabolism in tumor‐infiltrating CD8⁺ T and natural killer cells, restoring their durability and leading to complete local tumor eradication. This combination expands a distinct CD8⁺ T‐cell subset with *Tcf‐1*⁺ progenitor‐exhausted features and elevated memory/effector gene expression, enhancing proliferation and cytotoxicity, as shown by single‐cell RNA sequencing. Intranasal nanoSTING@Mn delivery mobilizes these LLL‐revived T cells to the lung, where they differentiate into resident memory T cells and establish systemic antitumor immunity. Upon intravenous rechallenge, disseminated tumor cells are eliminated, preventing metastasis and ensuring long‐term protection. This synergistic approach offers a scalable platform to boost immunotherapy efficacy and redefines immune‐based metastasis prevention strategies.

## Graphical abstract

Low‐level light (LLL) rejuvenates exhausted CD8+ T cells, boosting STING‐driven tumor eradication and prevents metastasis. NanoSTING@Mn enhances CD8+ T cells infiltration while LLL restores its function expanding to progenitor‐exhausted cells. These cells form central memory T cells in lymphoid organs. Intranasal nanoSTING@Mn redirects antitumor CD8+ T cells to lungs, where they form resident memory T cells blocking metastatic colonization

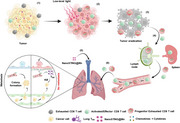

## 1 Introduction

Immunotherapy has transformed cancer treatment by offering unprecedented curative potential in malignancies once deemed refractory. Central to this paradigm shift are cytotoxic CD8⁺ T cells, which mediate durable tumor control through antigen‐specific recognition and destruction of malignant cells.^[^ ^1^ ^]^ Despite remarkable clinical successes, the majority of patients with solid tumors fail to achieve long‐term benefit from current immunotherapies, including immune checkpoint blockades (ICBs).^[^ ^2^, ^3^ ^]^ A major impediment to durable responses is the profound immunosuppressive tumor microenvironment (TME), marked by hypoxia, nutrient deprivation, and elevated oxidative stress. These adverse conditions disrupt the mitochondrial integrity of tumor‐infiltrating lymphocytes (TILs) under constant antigen stimulation.^[^ ^4^ ^]^ Mitochondrial dysfunction has emerged as a central driver of T‐cell exhaustion, a dysfunctional state characterized by diminished cytokine production, proliferation, and cytotoxicity.^[^ ^5^ ^]^ Exhausted T cells exhibit fragmented mitochondria, loss of membrane potential, and enhanced mitophagy, all of which compromise their bioenergetic fitness and immune surveillance.^[^ ^6^ ^]^ The metabolic dysfunction may also contribute to the disappointing outcomes of clinical trials with the stimulator of interferon genes (STING) agonists such as ADU‐S100.^[^ ^7^, ^8^ ^]^ Although intratumoral (IT) administration of the STING agonists rigorously activates innate immunity and enhances CD8⁺ T‐cell infiltration, it fails to sustain the antitumor responses.^[^ ^9^, ^10^ ^]^ Even in combination with ICBs (e.g., anti‐PD‐L1 or anti‐CTLA‐4), clinical benefits have been modest in patients with lymphomas and other solid tumors.^[^ ^11^ ^]^ These challenges underscore the urgent need to restore T‐cell metabolic fitness and overcome exhaustion to fully realize the potential of immunotherapy in solid tumors.

Mitochondrial fitness is the central determinant of T‐cell fates and function and remains a driving force for developing long‐lived memory T cells, while metabolic derangements, such as depolarization and impaired oxidative phosphorylation, are hallmarks of terminal T‐cell exhaustion.^[^ ^12^ ^]^ Therapeutic strategies aimed at enhancing mitochondrial fitness, such as enforced expression of fusion proteins,^[^ ^13^ ^]^ pharmacologic activation of biogenesis,^[^ ^14^ ^]^ or intercellular mitochondrial transfer^[^ ^15^ ^]^ have demonstrated promise in restoring T‐cell effector functions and reestablishing immune surveillance in preclinical tumor models. Recently, Baldwin et al. developed a novel platform that transferred healthy mitochondria from bone‐marrow‐derived stem cells into CD8⁺ T cells via tunneling nanotubes, thereby rescuing metabolic competence.^[^ ^15^ ^]^ Separately, pharmacological activation of pyruvate kinase M2 (PKM2) was shown to enhance CD8⁺ T‐cell antitumor function by promoting mitochondrial biogenesis, improving network organization, and restoring oxidative metabolism.^[^ ^14^ ^]^ Moreover, organelle crosstalk has gained attention as a critical axis of metabolic regulation. For instance, enforced expression of mitofusion‐2 in CD8⁺ TILs improved metabolic resilience and potentiated antitumor immunity.^[^ ^13^ ^]^ These studies highlight the compelling and vital therapeutic strategies by reprogramming mitochondrial metabolism in IT CD8^+^ T cells for effective immunotherapy.

The current investigation exploits low‐level light (LLL) therapy, a form of photo‐biomodulation, as an innovative, clinically practical, noninvasive, and drug‐free therapeutic to directly rewire mitochondrial functions in IT CD8^+^ T cells for effective immunotherapy. Unlike conventional pharmacologic agents that depend on systemic distribution and intact receptor signaling (often compromised within tumors), LLL directly enhances mitochondrial respiratory chain activity by activating complexes I, III, and IV of the electron transport chain, thereby converting light energy into bioenergy in the form of ATP within the mitochondrial inner membrane.^[^ ^16^, ^17^ ^]^ Because ATP production is light‐driven, it is relatively less dependent on oxygen and remains effective in ischemic or near‐hypoxic tissues, as demonstrated both in vivo^[^ ^18^, ^19^ ^]^ and in vitro.^[^ ^20^, ^21^ ^]^ This unique feature makes LLL particularly suited to stimulate mitochondrial biogenesis in IT CD8^+^ T cells, a well‐known hypoxic microenvironment. We also showed that 810 nm LLL upregulated the expression of peroxisome proliferator‐activated receptor‐gamma coactivator‐1α (PGC‐1α)—a master regulator of mitochondrial biogenesis—and its downstream targets.^[^ ^22^ ^]^ PGC‐1α plays a central role in cellular energy metabolism and promotes cell survival.^[^ ^23^ ^]^ A recent study showed that LLL enhanced mitochondrial functions of CD8^+^ T cells ex vivo and improved antitumor efficacy after their adoptive transfer compared to control T cells.^[^ ^24^ ^]^ It is worthwhile to mention that LLL does not enhance ATP production in cancer cells, which predominantly rely on aerobic glycolysis for energy. As such, LLL may represent a mechanistically distinct and clinically scalable modality to sustain T‐cell bioenergetics within tumors.

To explore the potential of LLL in immunotherapy, a robust tumor infiltration of CD8^+^ T cells was first stimulated by engineering nanoSTING@Mn, to address the inherent paucity of T cells in solid lymphoma. NanoSTING@Mn was fabricated by a biomimetic liposome encapsulating the STING agonist ADU‐S100 coordinated with manganese ions (Mn^2^⁺).^[^ ^10^, ^25^ ^]^ NanoSTING@Mn effectively orchestrated a robust innate immune response and promoted substantial CD8^+^ T‐cell infiltration into the tumor. However, nanoSTING@Mn alone proved insufficient for durable tumor control due to CD8^+^ T‐cell exhaustion. Remarkably, the T‐cell exhaustion was significantly rescued by LLL application, which promoted mitochondrial biogenesis, effector cytokine production, and sustained functionality of the cells within the tumor. As a result, the combined therapy not only eradicated lymphoma but also established durable memory antitumor immunity, which could be redeployed to the lungs by intranasal nanoSTING@Mn delivery, resulting in complete prevention of systemic tumor rechallenge and metastasis. Single‐cell RNA sequencing (scRNA‐seq) revealed a unique enrichment of a transcriptionally distinct CD8^+^ T progenitor‐exhausted (Tpex) cells, characterized by elevated metabolic and cytotoxic signatures and expressing *Tcf‐1* ^+^ progenitor‐exhausted features, concurrent with robust memory T‐cell development. Apart from CD8^+^ T cells, LLL also enhanced the fitness and functionality of natural killer (NK) cells. The investigation demonstrates that integrating photonic energy with innate immune activation can synergistically overcome the metabolic barriers, reviving CD8^+^ T‐ and NK‐cell efficacy in solid tumors.

## 2 Results

### 2.1 Activation of the cGAS–STING Pathway to Stimulate Tumor Infiltration of CD8^+^ T Cells

**Figure 1:**
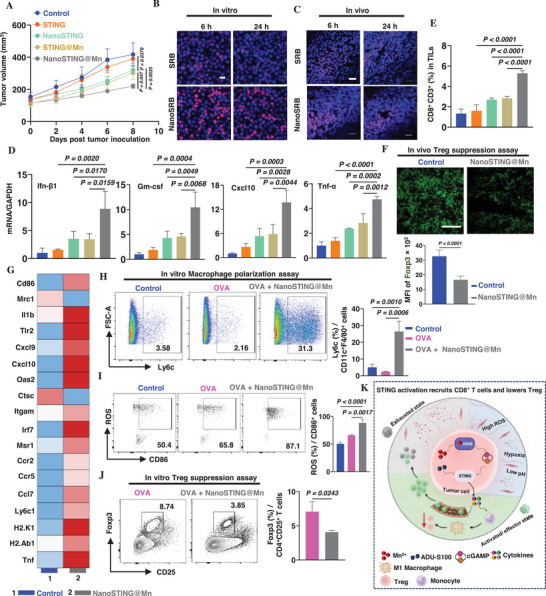
cGAS–STING activation with nanoSTING@Mn recruits CD8^+^ T cells and modulates tumor immunity. A) C57BL/6 mice were subcutaneously inoculated with 1 × 10⁶ EL4 tumor cells and treated intratumorally (i.t.) with STING (red), nanoSTING (cyan blue), STING@Mn (brown), or nanoSTING@Mn (gray), each containing 5 µg of ADU‐S100 on days 0 and 3 after tumors reached 100 mm^3^. Tumor volumes were monitored every two days until day 8. B) Internalization of SRB and nanoSRB was visualized in EL4 cell culture at 6 and 24 h under confocal microscopy. C) SRB distribution was tracked in 6 and 24 h after i.t. injection of free SRB or nanoSRB. D) Cytokine expressions in tumor tissues were assessed 24 h after the second dose of i.t. administration of indicated STING agonist formulations by qRT‐PCR. E) Flow cytometric analysis showed increased CD8⁺ T‐cell infiltration in tumors treated with nanoSTING@Mn on day 8. F) Foxp3⁺ regulatory T cells (Tregs, green) decreased on day 12 after initial treatment as shown by immunofluorescent staining of tumor sections (upper) and flow cytometry (bottom). G) Heatmap of monocyte reprogramming genes 12 days after the initial nanoSTING@Mn treatment, from scRNA sequencing of TILs. Control (blue, 1), nanoSTING@Mn (gray, 2), H,I) Mouse bone marrow cells differentiated into *Ly6c^+^* macrophages identified as *Ly6c^+^* CD11c^+^F4/80^+^ (H), and expressed ROS as stained with Cellrox in M1 macrophages CD86^+^Ly6c^+^CD11c^+^F4/80^+^ (I), following stimulation with ovalbumin (OVA), a model antigen, in the presence or absence of nanoSTING@Mn. Representative flow cytometric profiles are shown on the left, and summary results are shown on the right. J) Suppression of Treg‐cell differentiation by nanoSTING@Mn‐induced M1 macrophages. Mouse bone marrow cells differentiated in the presence of nanoSTING@Mn as (H) and (I) were cocultured with CD4^+^ T cells for 6 days, and Foxp3^+^ in CD4^+^CD25^+^ were analyzed by flow cytometry. K) Mechanism of antitumor effect with i.t. administration of nanoSTING@Mn, created by Biorenders.com. The data are expressed as mean ± SEM and were analyzed by one‐way analysis of variance (ANOVA) (D, E, H, and I), unpaired *t*‐test (F and J), and two‐way ANOVA (A) followed by post‐hoc Tukey's test for multiple comparisons. *n* = 5 for (A), 3 for (B, D–F, H–J), 4 for (C). Scale bar, 20 µm for (B), 40 µm for (C), and 80 µm for (F). Representative of two independent experiments.

Both preclinical^[^ ^26^ ^]^ and clinical observations^[^ ^27^ ^]^ have shown that treatment with PD‐1 and PD‐L1 blocking antibodies promotes progression of T‐cell lymphoma, raising serious concerns.^[^ ^24^, ^28^ ^]^ The potential harm of PD‐1 blockade in T‐cell lymphoma was further corroborated by a phase 2 clinical trial for adult T‐cell lymphoma, where 3 patients experienced rapid disease progression after a single dose of the PD‐1 inhibitor nivolumab.^[^ ^29^ ^]^ These observations underscore the urgent need for alternative approaches to treat T‐cell lymphoma. Accordingly, T‐cell lymphoma was selected as a solid tumor model to investigate the beneficial effect of LLL on CD8^+^ T cells. However, given the inherent paucity of functional CD8^+^ T cells in solid T‐cell lymphoma, direct LLL application is unlikely to be effective. Previous investigation showed that ADU‐S100 complexed with Mn was the most potent activator for the cGAS–STING pathway,^[^ ^10^ ^]^ wherein ADU‐S100 directly activates STING, while Mn has been shown to enhance cGAS activity independently of double‐stranded DNA.^[^ ^30^, ^31^ ^]^ Moreover, a phase I clinical trial by Lv et al. revealed that Mn promoted robust type I interferon (IFN) production, leading to safe and significant antitumor immune responses in patients with advanced metastatic solid tumors.^[^ ^32^ ^]^ The dual activator STING@Mn resulted a significant reduction in the tumor volume compared to ADU‐S100 alone, when measured on day 8 after IT administration twice a week (1.2‐fold; *p* = 0.0379, **Figure** **1**A). We then encapsulated STING@Mn into biomimetic liposomes, forming nanoSTING@Mn to enhance the intracellular delivery.^[^ ^10^, ^33^ ^]^ To verify the intracellular delivery by nanoSTING@Mn, sulforhodamine B (SRB), a fluorescent dye with a size and charge similar to ADU‐S100, was encapsulated into the biomimetic liposomes, forming SRB‐loaded nanoparticles (nanoSRB). Incubation of nanoSRB with EL4 cells in cultures for varying times showed substantial increases in intracellular SRB fluorescence at 6 and 24 h compared to free SRB (Figure 1B). Similar results were also attained in vivo, proving superior retention of nanoSRB inside tumor cells compared to free SRB after IT administration (Figure 1C).

The efficient intracellular delivery of STING@Mn greatly increased its antitumor efficacy, as evidenced by a more significant decrease in the tumor volume when nanoSTING@Mn was IT administered in comparison with STING@Mn (1.3‐fold; *p* = 0.047). The presence of Mn was significant since ADU‐S100 alone encapsulated within the biomimetic liposomes was not as sufficient as nanoSTING@Mn, showing 1.4‐fold inferiority (*p* = 0.0035). The superior antitumor effect of nanoSTING@Mn was consistent with robust increases in induction of *Ifn‐β1* (2.5‐fold; *p* = 0.0159), *Gm‐csf* (2.2‐fold; *p* = 0.0068), *Cxcl10* (2.3‐fold; *p* = 0.0044), and *Tnf‐α* (1.6‐fold; *p* = 0.0012) compared to STING@Mn or nanoSTING (Figure 1D). A significant increment of CD8^+^ T‐cell infiltration into the tumor was also seen on day 8 following treatment with nanoSTING@Mn compared to STING alone (3.2‐fold; *p* < 0.0001) or nanoSTING (1.9‐fold; *p* < 0.0001) (Figure 1E). Representative flow cytometric gating and profiles are shown in Figure S1 (Supporting Information). This finding validates that the complexation of STING agonists with Mn^2^⁺, coupled with their encapsulation within a nanosystem, significantly enhances antitumor immune responses by facilitating tumoral infiltration of CD8^+^ T cells.

#### 2.1.1 STING Activation Instructs M1‐Macrophage Polarization and Downregulates Regulatory T (Treg) Cells

Apart from robust infiltration of CD8^+^ T cells, STING activation was found to suppress Treg cells, shifting an immunosuppressive TME from “cold” to “hot” one. As shown in Figure 1F, Foxp3 expression, a surrogate biomarker for Treg cells, was expressed highly as shown by bright immune fluorescent staining (upper) and flow cytometric analysis of Foxp3^+^ T cells (bottom), yet diminished substantially compared to controls by day 12 after IT administration of nanoSTING@Mn. The effect may be attributed to the increased production of type I interferons, such as *Ifn‐β*, which are known to polarize tumor‐associated monocytes toward an immunostimulatory state and inhibit Treg cells.^[^ ^34^ ^]^ To investigate this, we performed scRNA‐seq of TILs on day 12. As shown in the heatmap in Figure 1G, monocyte‐associated genes such as *Ly6c1* and *Ccr2* were significantly upregulated in the nanoSTING@Mn‐treated group, indicating an influx of circulating monocytes.^[^ ^35^ ^]^ The infiltrated monocytes differentiated predominantly toward an M1‐like macrophage phenotype, as suggested by elevated expression of M1‐associated genes, including antigen presentation markers (*H2‐Ab1*, *H2‐K1*, *Cd86*), inflammatory chemokines (*Ccl7*, *Cxcl9*, *Cxcl10*), chemokine receptors (*Ccr2*, *Ccr5*), and interferon‐stimulated genes (*Oas2*, *Irf7*). In marked contrast, M2‐macrophage‐related genes such as *Mrc1* and *Ctsc* were notably downregulated.^[^ ^36^ ^]^

To verify whether nanoSTING@Mn modulates Treg cells in TME through M1‐polarized macrophages, monocytes generated from bone marrow cells were stimulated by ovalbumin (OVA) model antigen in the presence or absence of nanoSTING@Mn. The presence of nanoSTING@Mn greatly elevated the differentiation of *Ly6c^+^* macrophages compared to the OVA (10.8‐fold; *p* = 0.0006) alone or controls (5.2‐fold; *p* = 0.001, Figure 1H). These *Ly6c^+^* macrophages expressed a higher level of reactive oxygen species (ROS), a hallmark of M1 macrophage phenotype relatively to OVA alone (1.3‐fold; *p* = 0.0017) or controls (1.7‐fold; *p* < 0.0001) (Figure 1I). Functionally, these macrophages suppressed Treg‐cell differentiation by more than 40% when cocultured with CD4^+^ T cells for 6 days (1.7‐fold; *p* = 0.0243) (Figure 1J). Taken together, nanoSTING@Mn not only vigorously recruits CD8^+^ T cells but also suppresses Treg cells, reshaping the tumor immune landscape. However, despite this immunologic remodeling, CD8⁺ T cells exhibited poor persistence, became functionally exhausted within the hostile TME (Figure 1K), and failed to control tumor growth (Figure 1A). This underscores the critical need for strategies that not only recruit but also preserve and potentiate the functionality of IT CD8⁺ T cells to achieve durable tumor control.

#### 2.1.2 LLL Sustains the Functionality and Number of IT CD8^+^ T Cells, Leading to Tumor Eradication

**Figure 2:**
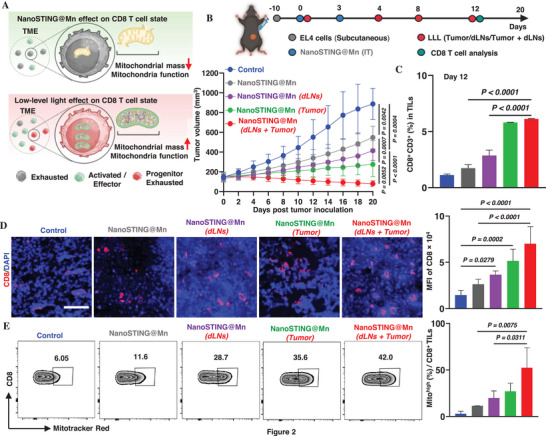
NanoSTING@Mn combined with LLL eradicates tumors. A) The schematic illustrates the potential of LLL to enhance the functionality of i.t. CD8^+^ T cells for strong antitumor effects, created by Biorenders.com. B) LLL and nanoSTING@Mn synergistically eradicate tumors. EL4 cells were inoculated and grew into tumors as Figure 1B. Tumor‐bearing C57BL/6 mice were i.t. treated with nanoSTING@Mn containing 5 µg of ADU‐S100 on days 0 and 3. LLL was applied onto the tumor, dLNs, or both on days 0, 4, 8, and 12, and tumor volume was monitored over time. Some tumors were analyzed on day 12 after the initial treatment (day 0) for C) CD8⁺ T‐cell infiltration and E) mitochondrial mass (Mitotracker red^+^ CD3^+^CD8^+^) by flow cytometry or confocal images of CD8 (red) and D) nuclei (DAPI, blue). Representative images or flow cytometric profiles are shown on the left, and the corresponding statistical analyses are shown on the right in (D and E). The data are expressed as mean ± SEM and were analyzed by one‐way (C–E) ANOVA and mixed‐effect (B) analysis followed by post‐hoc Tukey's test for multiple comparisons. *n* = 10 for (B), 3 for (C–E). Scale bar, 80 µm for (D). Representative of two independent experiments. In the labels, dLNs and Tumor shown in red indicate groups that received LLL treatment.

Our preliminary antitumor study evaluating the effect of LLL treatment alone in the EL4 tumor model showed that LLL by itself did not show antitumor effect and tumor volume was comparable to untreated controls, suggesting that LLL does not exert a direct antitumor effect (Figure S2a, Supporting Information). The detailed method and result is provided in the Supporting Information. Thus, here we hypothesized that LLL could sustain the functionality of IT CD8^+^ T cells and unlock the full potential of these IT CD8^+^ T cells in tumor eradication, as shown in **Figure** **2**A. Accordingly, we applied 810 nm LLL directly and noninvasively at a power density of 3 J cm^−^^2^ to the tumor, draining lymph nodes (dLNs), or both, because the immune suppressive TME influences dLNs adversely, and the two were mutually influenced. The functional state of immune cells in the dLNs can influence the composition and activity of infiltrating lymphocytes within the tumor. Thus, the TME and dLNs are dynamically interconnected, with reciprocal interactions shaping the magnitude and quality of the antitumor immune response. By targeting both sites, we believe that LLL can simultaneously enhance T‐cell priming in dLNs and sustain effector function within the tumor, thereby improving overall antitumor immunity.^[^ ^37^, ^38^, ^39^ ^]^ As shown in Figure 2B, a combination of IT nanoSTING@Mn and LLL illumination of the tumor led to a 3.2‐fold reduction in tumor volume by day 20 as compared to controls (*p* < 0.0001) or a twofold decrease compared to nanoSTING@Mn alone (*p* = 0.0007). The reduction was furthered when LLL was applied to both tumors and dLNs, giving rise to a 5.2‐fold decrease in tumor growth compared to nanoSTING@Mn (dLNs) (Figure 2B). Although some residual masses were still measurable in mice treated with nanoSTING@Mn (dLNs + Tumor) regimen on day 20, no tumor growth was observed over the subsequent 70 days survival study (Figure 5F), suggesting that the resident masses were likely necrotic debris rather than viable tumor cells. Thus, the dual treatment led to complete tumor regression. Notably, no significant changes in body weight (Figure S2d, Supporting Information) and 100% survival rate (Figure S2e, Supporting Information) were observed in the LLL and nanoSTING@Mn treatment group compared to controls, confirming that the treatment was well tolerated and nontoxic.

Flow cytometric analyses of TILs on day 12 posttreatment verified ≈50% decline in CD8^+^ T‐cell frequencies (1.7 ± 0.3%; Figure 2C) relative to day 8 (5.3 ± 0.2%; Figure 1E) in the absence of LLL, in agreement with transient effector functionality. In sharp contrast, LLL targeting both dLNs and the tumor sustained nanoSTING@Mn‐mediated CD8^+^ T‐cell infiltration, yielding a 3.5‐fold increase (*p* < 0.0001) in the presence versus absence of LLL (Figure 2C). Flow cytometric gating and representative profiles are shown in Figure S2b (Supporting Information). The ability to sustain IT CD8^+^ T cells at consistent levels through the immunotherapy highlights the critical role of LLL in achieving durable antitumor immunity. These results validate the potential of nanoSTING@Mn combined with LLL to synergistically retain effector T cells and enhance therapeutic outcomes.

The increases in the number of IT CD8^+^ T cells were further investigated by immunofluorescence staining of tumor tissues. CD8^+^ T‐cell accumulation in the tumor (left) and the mean fluorescence intensity (MFI) (right) were increased significantly by LLL illumination on either tumor or dLNs, with the greater increase observed when LLL was exposed to both (Figure 2D). Remarkably, mitochondrial mass of IT CD8^+^ T cells increased by 4.5‐fold with LLL applied to both dLNs and the tumor as compared to its absence (*p* < 0.05, Figure 2E), as revealed by Mitotracker Red staining on gated CD8^+^ T cells (Figure S2c, Supporting Information). These results validate that LLL exposure to both dLNs and tumor enhances the functionality of tumor‐infiltrating CD8^+^ T cells, thereby providing a compelling rationale for the combined use of LLL with nanoSTING@Mn to achieve a potent antitumor immunity (Figure 2A).

#### 2.1.3 LLL Upregulates Gene Expressions Associated with CD8^+^ T‐Cell Functionality

**Figure 3:**
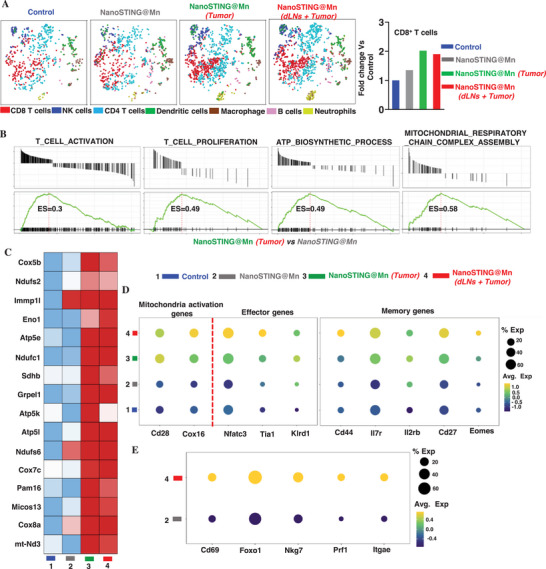
ScRNA sequencing of TILs identifies genes upregulated by LLL, in association with CD8^+^ T‐cell functionality. Tumor inoculation and treatment were performed as Figure 2B, and TILs were isolated and subjected to scRNA sequencing on day 12 after initial treatment. A) Cell type annotation demonstrates preferential enrichment of CD8^+^ T cells, NK cells, and neutrophils in the presence compared to the absence of LLL treatment (left). Notably, CD8^+^ T cells were doubled with LLL treatment (right). B) Gene set enrichment analysis (GSEA) shows increased expression of genes associated with activation, proliferation, ATP biosynthesis, and mitochondrial activity in T cells between nanoSTING@Mn–LLL on Tumor (green) and nanoSTING@Mn alone (gray). C) Heatmap analysis of mitochondrial‐associated genes essential for ATP biosynthesis and OXPHOS, which were upregulated by LLL. D) Dot‐plot analysis of LLL‐mediated upregulation of genes associated with mitochondrial activity (*Cd28*, *Cox16*), cytotoxic function (*Nfatc3*, *Tia1*, *Klrd1*), and memory‐associated markers (*Cd44*, *Il7r*, *Il2rb*, *Cd27*, *Eomes*) in CD8⁺ T cells. E) Expanded dot plots show LLL‐driven upregulation of key genes related to CD8^+^ T‐cell activation (*Cd69*), mitochondrial biogenesis and metabolic regulation (*Foxo1*), cytotoxicity (*Prf1*, *Nkg7*), and tissue residency/memory (*Cd103*), compared to the group without LLL. Control (blue, 1), nanoSTING@Mn (gray, 2), nanoSTING@Mn (Tumor) (green, 3), nanoSTING@Mn (dLNs + Tumor) (red, 4) in (C–E).

Having established the potent antitumor effects of LLL–nanoSTING@Mn combination, we went on to determine the gene profile associated with TIL differentiation by scRNA sequencing on day 12 after the dual treatment. Seven main cell clusters were analyzed, including CD8^+^ T cells, NK cells, CD4^+^ T cells, dendritic cells, macrophages, B cells, and neutrophils (**Figure** **3**A, left panel). CD8^+^ T cells and to a lesser extent, NK cells were enriched considerably with LLL treatment than without it (Figure 3A). Quantitatively, CD8^+^ T cells increased by more than twofold with LLL illumination compared to control (Figure 3A, right panel). Gene set enrichment analysis (GSEA) demonstrated upregulation of several gene clusters related to T‐cell activation and proliferation in the presence compared to the absence of LLL (Figure 3B). Interestingly, compared to nanoSTING@Mn group, LLL treatment remarkably restored the expression of genes associated with mitochondrial activities and respiratory chain complex (Figure 3B). This mitochondrial rescue is particularly significant, as mitochondrial dysfunction has been implicated in the exhaustion and metabolic collapse of CD8⁺ T cells in tumors.^[^ ^5^, ^40^ ^]^ In the nutrient‐deprived and immunosuppressive TME, decreased mitochondrial membrane potential, coupled with sustained PD‐1 expression, leads to impaired OXPHOS, reduced ATP production, and ultimately compromised antitumor activity.^[^ ^41^ ^]^ Heatmap analysis revealed elevated expression of several mitochondrial genes essential for ATP biosynthesis and OXPHOS in Figure 3C. This included cytochrome c oxidase subunits (*Cox5b*, *Cox7c*, *Cox8a*), ATP synthase components (*Atp5e*, *Atp5l*), and NADH dehydrogenase subunits (*Ndufs2*, *Ndufc1*, *Ndufs6*, *mt‐Nd3*).^[^ ^42^, ^43^ ^]^ In addition, the succinate‐dehydrogenase‐associated gene *Immp1l* was also upregulated. The coordinated upregulation of these genes reaffirms a comprehensive metabolic reprogramming, by LLL, of CD8⁺ T cells toward a highly oxidative phenotype capable of sustaining ATP production under nutrient‐limited and hypoxic conditions.

By restoring mitochondrial integrity and OXPHOS activity, LLL revived CD8⁺ T‐cell energy metabolism, contributing to improved persistence and antitumor efficacy substantially (Figure 2B). Dot plot analysis of differentiated gene expression confirmed a high proportion of CD8^+^ T cells in tumor following dual LLL–nanoSTING@Mn treatment, with elevated expressions of genes associated with mitochondrial activation (*Cd28*, *Cox16*),^[^ ^44^ ^]^ memory function (*Cd44*, *Il7r*, *Il2rb*, *Cd27*, *Eomes*),^[^ ^45^ ^]^ and cytotoxic effector function (*Nfatc3*, *Tia1*, *Klrd1*) (Figure 3D).^[^ ^45^, ^46^, ^47^ ^]^ By contrast, treatment with nanoSTING@Mn alone did not enhance the expression of these genes as compared to the controls. Encouraged by this exciting result, we further compared the gene expression pattern between nanoSTING@Mn and LLL–nanoSTING@Mn (dLNs + Tumor). Additional genes *Cd69*, *Foxo1*, *Nkg7*, *Prf1*, *Itgae* were also increased in a high proportion of CD8^+^ T cells in connection with their activation, mitochondrial activity, memory, and function in the presence versus absence of LLL (Figure 3E). The molecular imprint underscores the profound impact of LLL on the potentiation of CD8^+^ T cells.

#### 2.1.4 LLL Predominantly Augments Progenitor‐Exhausted CD8^+^ T Cells

**Figure 4:**
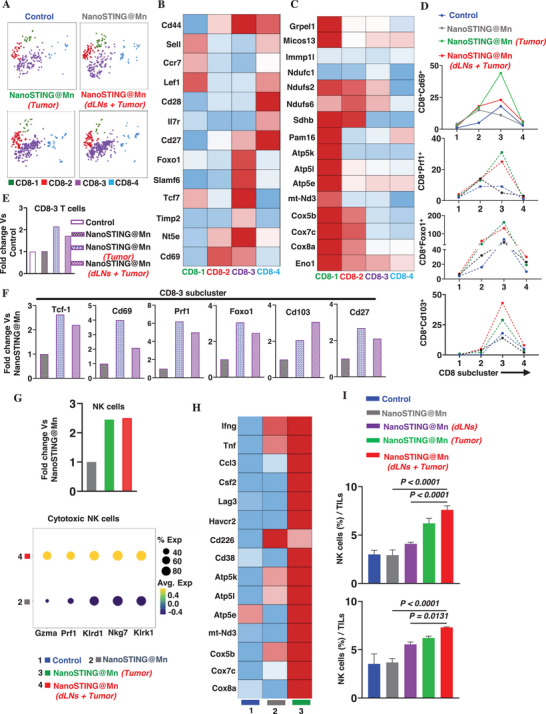
LLL substantially enhances the function and proportion of CD8^+^ progenitor‐exhausted T cells (Tpex). A) Network‐based clustering of CD8+ T cells into four distinct subclusters: CD8‐1 (green), CD8‐2 (red), CD8‐3 (purple), and CD8‐4 (blue). B) Heatmap shows distinct transcriptional imprints of these CD8⁺ T‐cell clusters, aligning CD8‐1 with naïve, CD8‐2 with activated, CD8‐3 with progenitor exhausted Tpex, and CD8‐4 with memory T cells. C) Heat map also shows that the CD8‐4 subset expresses high levels of genes involved in ATP biosynthesis and OXPHOS, a characteristic feature of memory T cells. D,E) LLL leads to a twofold increase in Tpex (CD8‐3) subcluster (E), characterized by robust upregulations of activation (*Cd69* ^+^CD8‐3^+^), cytotoxicity‐associated (*Prf1* ^+^CD8‐3^+^), mitochondrial fitness (*Foxo1* ^+^CD8‐3^+^), and tissue‐resident (*Cd103* ^+^CD8‐3^+^) markers (D). F) CD8‐3 subcluster expresses surrogate markers of progenitor‐exhausted T cells (*Tcf‐1*), as well as genes associated with T‐cell activation (*Cd69*), mitochondrial function (*Foxo1*), cytotoxicity (*Prf1*), and memory/survival (*Cd103*, *Cd27*). G) LLL also enhances the functional activity of NK cells as evidenced by more than twofold increases in the frequency of NK cells in the presence versus the absence of LLL (Upper). Dot plot analysis reveals that LLL upregulates gene signatures associated with NK‐cell cytotoxicity and activation (*Gzma*, *Prf1*, *Klrd1*, *Nkg7*, and *Klrk1*) (bottom). H) Heat map indicates that LLL upregulates key genes that support NK‐cell maturation and activation. I) Flow cytometric analysis of NK cells marked as CD3^−^NK1.1^+^ infiltration of tumor tissues at days 12 (top) and 20 (bottom) posttreatment. The data were analyzed by one‐way ANOVA. *n* = 3 for (I). Control (blue, 1), nanoSTING@Mn (gray, 2), nanoSTING@Mn (Tumor) (green, 3), nanoSTING@Mn (dLNs + Tumor) (red, 4) in (G) and (H).

To delineate the CD8^+^ T‐cell subset most responsive to LLL, we leveraged unsupervised network‐based clustering and identified four transcriptionally distinct CD8^+^ T‐cell subclusters, designated as CD8‐1 (green), CD8‐2 (red), CD8‐3 (purple), and CD8‐4 (blue), across different samples (**Figure** **4**A). CD8‐1 subset expressed high levels of naïve T‐cell markers (*Sell*, *Ccr7*, *Lef1*),^[^ ^48^ ^]^ representing naïve CD8 T cells (Figure 4B). CD8‐2 likely corresponded to activated CD8^+^ T cells, characterized by upregulation of Cd44 expression and low levels of *Ccr7 and Il7r* (Figure 4B). CD8‐3 expressed hallmark biomarkers of Tpex CD8^+^ T cells, including *Slamf6*, *Tcf7*, along with upregulated TGFβ signaling genes (*Timp2*, *Nt5e)*, a signature feature of Tpex CD8^+^ T cells (Figure 4B).^[^ ^49^ ^]^ CD8‐4 cells showed high levels of *Cd44 and Il7r* expression, along with *Ccr7 and Sell*, consistent with a central memory T‐cell phenotype^[^ ^50^ ^]^ (Figure 4B). Furthermore, CD8‐4 cells also exhibited the highest expression of genes involved in OXPHOS, including *Ndufc1*, *Ndufs2*, *Ndufs6*, *mt‐Nd3*, *Cox5b*, *Cox7c*, *Cox8a*, and ATP biosynthesis‐related genes (*Atp5k*, *Atp5l*, *Atp5e*)^[^ ^42^, ^43^ ^]^ (Figure 4C). This metabolic profile is characteristic of memory T cells, which require sustained energy production to support their long‐term survival and rapid effector responses whenever needed. By contrast, the naïve‐like CD8‐1 subset minimally expressed these metabolic genes (Figure 4C). We also conducted pseudotime trajectory plot analysis which also indicates a consecutive differentiation progress of CD8 T cells after treatment, by which activated CD8 T cells (CD8‐2, red) were differentiated into Tpex cells (CD8‐3, purple), and further mature into memory T cells (CD8‐4, blue) (Figure S3a, Supporting Information).

Among the four clusters, CD8‐3 emerged as the primary target of LLL‐mediated enrichment of T cells. The dual treatment with LLL and nanoSTING@Mn enhanced the abundance of the CD8‐3 cluster by 1.7‐fold compared to either control or nanoSTING@Mn alone (Figure 4E). While nanoSTING@Mn alone preferentially induced the CD8‐2 cluster, as evidenced by elevated expression of activation marker *Cd69* (threefold) and cytotoxic effector, perforin (*Prf1*, 1.5‐fold) (Figure 4D). This activation was transient and insufficient to sustain effector function or survival, likely due to the hypoxic and immunosuppressive TME (Figure 4D). By contrast, CD8‐3 cluster was characterized by a robust upregulation of both activation and cytotoxicity‐associated markers, with *Cd69* and *Prf1* expression increasing by 2.4‐fold and 3.4‐fold, respectively, compared to the control (Figure 4D). These results suggest that LLL not only enhances initial activation but also considerably mitigates the rapid attrition of effector T cells typically observed with treatment with STING agonist alone. Given the central role of mitochondrial fitness in sustaining T‐cell functionality and memory formation, we next evaluated the expression of *Foxo1*, a key transcriptional regulator of mitochondrial biogenesis and metabolic homeostasis.^[^ ^5^ ^]^ *Foxo1* enhances mitochondrial biogenesis and metabolic capacity, which is essential for increasing the mitochondrial capacity critical for the development and persistence of memory T cells.^[^ ^51^, ^52^ ^]^ LLL treatment significantly increased *Foxo1* expression across all four CD8 clusters, with the most pronounced effect on CD8‐3 cluster, a threefold increase compared to nanoSTING@Mn, which alone failed to increase *Foxo1* expression over the controls (Figure 4D). These findings suggest that LLL promotes mitochondrial biogenesis, thereby enhancing their longevity and metabolic adaptability. Moreover, CD8‐3 cells also expressed a high level of *Cd103*, a defining marker of tissue‐resident memory T (T_RM_) cells known to mediate durable local immune surveillance. Notably, *Cd103* expression increased by threefold within CD8‐3 T cells following nanoSTING@Mn + LLL treatment, whereas other CD8 clusters showed no significant changes (Figure 4D).

Importantly, CD8‐3 cluster expressed *Tcf‐1*, a key transcription factor essential for self‐renewal and memory‐like features, and a hallmark of Tpex cells (Figure 4F). These T cells serve as a stem‐like reservoir that sustains antitumor immunity through their proliferative potential and capacity to differentiate into effector and memory T cells.^[^ ^53^ ^]^ A great number of studies have shown positive correlation of their presence in tumors with better therapeutic outcomes.^[^ ^54^, ^55^ ^]^ Consistent with the Tpex phenotype, genes associated with activation (*Cd69*), mitochondrial function (*Foxo1*), cytotoxicity (*Prf1*), and memory/survival (*Cd103*, *Cd27*) were enriched within this cluster, reinforcing the conclusion that LLL acts as a metabolic and functional amplifier of IT CD8^+^ T cells, especially to selectively expanding Tpex cells for durable tumor immunity. Notably, the ability of LLL to expand the Tpex pool mirrors the effects of ICB immunotherapy.^[^ ^55^ ^]^ However, LLL achieves this via localized and noninvasive treatment, effectively avoiding immune‐related adverse events commonly associated with systemic ICB immunotherapy.^[^ ^56^ ^]^ Furthermore, it circumvents the concern about tumor progression risk linked to anti‐PD‐1 treatment of T‐cell lymphoma.^[^ ^28^, ^57^ ^]^ Thus, LLL offers a distinct therapeutic advantage in the treatment of cutaneous T‐cell lymphoma.

#### 2.1.5 LLL Enhances NK‐Cell Functionality

Apart from CD8^+^ T cells, LLL also increased NK cells inside the tumor compared to the controls or IT nanoSTING@Mn, as revealed by scRNA sequencing (Figure 3A). Quantitatively, NK cells increased by ≈2.5‐fold with LLL illumination compared to nanoSTING@Mn (Figure 4G, top). Although unexpected, this finding is not entirely surprising, as LLL has been shown to preferentially enhance mitochondrial function in cells with high energy demands, such as neurons,^[^ ^16^ ^]^ muscles,^[^ ^19^ ^]^ and megakaryocytes.^[^ ^22^ ^]^ Both CD8^+^ T cells and NK cells are cytotoxic cells requiring high energy for cytotoxic activity. To gain further insight into the cytotoxic function of NK cells, we compared cytotoxic genes in the presence or absence of LLL. Gene signatures associated with NK‐cell cytotoxicity and activation (*Gzma*, *Prf1*, *Klrd1*, *Nkg7*, *and Klrk1*) were markedly upregulated at high proportions of NK cells in nanoSTING@Mn combined with LLL on dLNs and Tumor, as compared to nanoSTING@Mn alone (Figure 4G, bottom). Heatmap analysis revealed that while nanoSTING@Mn alone induced expression of *Ifng* and *Tnfa*, as well as chemokines (*Ccl3*) and growth factors (*Csf2*), it was insufficient to fully promote NK‐cell maturation, as suggested by low levels of expressing canonical maturation markers (*Lag3*, *Havcr2*) and modest upregulation of activation markers (*Cd226*, *Cd38*). By contrast, LLL treatment not only amplified expression of *Ccl3* and *Csf2* substantially, supporting enhanced recruitment and activation of innate immune cells, but also uniquely promoted NK‐cell maturation and activation programs.^[^ ^58^ ^]^ Furthermore, akin to its effects on CD8⁺ T cells, LLL markedly enhanced mitochondrial gene signatures in NK cells, indicating a fundamental shift toward a metabolically active, cytotoxic phenotype (Figure 4H).

We next evaluated CD3^−^NK1.1^+^ cells in tumors by flow cytometry over time, as gated in Figure S3b (Supporting Information), on days 12 (top) and 20 (bottom) after the dual treatment (Figure 4I). In alignment with scRNA sequencing data, percentages of NK cells were increased significantly by LLL applied on dLNs and tumors on both day 12 (2.6‐fold; *p* < 0.0001) and day 20 (1.9‐fold; *p* < 0.0001) compared to sham light (Figure 4I). These findings position LLL as an innovative approach to enhance the cytotoxic function of NK cells and support more robust antitumor responses.

### 2.2 NanoSTING@Mn Pulls Functional CD8^+^ T Cells and Prevents Lung Metastases

Successful anticancer therapy demands more than just the eradication of the primary tumor, as metastasis to secondary sites remains a major clinical challenge to manage. The lungs are a primary site of metastases in more than 20% of malignancies,^[^ ^59^, ^60^ ^]^ including in the EL4 lymphoma model. Our investigation showed that IT CD8^+^ T cells retained mitochondrial functional integrity with LLL treatment, accompanied by increasing central memory‐like CD8^+^CD44^+^ T cells. To harness this potential, we sought to deploy these effector/memory CD8^+^ T cells to the lungs to proactively prevent lung metastasis, an approach that has never been successfully attempted owing to its dependence on often poorly defined tumor antigens.^[^ ^61^ ^]^ Intriguingly, intranasal nanoSTING@Mn was found capable of pulling antigen‐primed T cells into the lung in an antigen‐independent manner, opening a new avenue for metastasis prevention.

**Figure 5:**
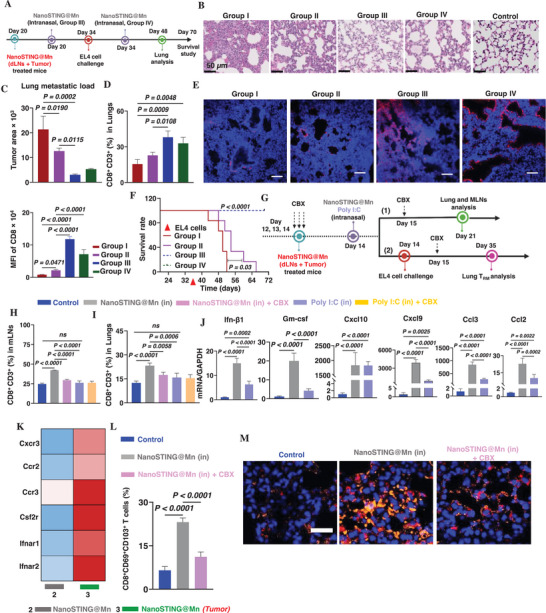
Intranasal administering NanoSTING@Mn pulls functional CD8^+^ T cells and prevents lung metastases. A) A schematic depicts the treatment and tumor rechallenge timeline. Tumor‐cured mice that had survived after 20 days of i.t. nanoSTING@Mn and LLL treatment (as Figure 2B) were intranasally administered nanoSTING@Mn either 14 days before (Group III) or on the same day as (Group IV) tumor rechallenge by intravenous injection of 1 × 10^5^ EL4 cells per mouse. Group I (naïve mice) and Group II (tumor‐cured mice without intranasal nanoSTING@Mn) were similarly challenged and served as controls for tumor growth and systemic immunity, respectively. B) Lung tissue was examined at day 48, and shown are representative H&E staining and histology quantification, C) lung metastatic load determined with Image J software, D) flow cytometry analysis of CD8^+^CD3^+^ T‐cell percentages in lung tissue, and E) immunofluorescent staining of CD8^+^ T cells (red) and DAPI (blue) (right) and statistical analysis of the immunofluorescent data (E, left). F) Kaplan–Meier survival curves of the treatment groups up to 70 days following initial treatment. G) A schematic depicts experiments aimed at determining the mechanism underlying intranasal nanoSTING@Mn‐mediated metastatic prevention. Mice treated with i.t. nanoSTING@Mn and LLL for 14 days (as Figure 2B) were administered either nanoSTING@Mn or poly I:C intranasally in the presence or absence of CBX. CBX was i.p. administered for four consecutive days, starting two days before (days 12 and 13) and continuing one day after (day 15) intranasal adjuvant administration. The dual‐treated mice without intranasal adjuvant administration served as systemic immunity controls (Control, blue). CD8^+^CD3^+^ T‐cell percentages in H) mLNs and I) lungs were evaluated by flow cytometry on day 21. J) Chemokine and cytokine expression in the lung tissue 16 h after the intranasal nanoSTING@Mn (gray) and Poly I:C (purple) over naïve controls (blue) by qRT‐PCR. K) Heatmap shows LLL‐mediated upregulation of chemokine and cytokine receptors in IT CD8^+^ T cells; nanoSTING@Mn (gray, 2), nanoSTING@Mn (Tumor) (green, 3). L) The percentage of lung T_RM_ CD8^+^ cells identified as CD3^+^CD8β^−^CD69^+^CD103^+^ cells was increased substantially by LLL, but nearly completely diminished in the presence of CBX treatment when examined on day 35 (depicted in G). These dual‐treated mice (as Figure 2B) were intravenously rechallenged with EL4 cells on day 14 as above (G), concurrent with intranasal nanoSTING@Mn with or without CBX. Dual‐treated mice without intranasal nanoSTING@Mn but with tumor rechallenge served as controls (blue). M) Representative immunofluorescence staining of lung T_RM_ cells on day 35 was conducted using confocal microscopy, with CD8 staining in orange, CD103 in red, and counterstaining with DAPI (blue). The data are expressed as mean ± SEM and were analyzed by one‐way (C, D, H–J, L) ANOVA, followed by post‐hoc Tukey's test for multiple comparisons and log‐rank test (F). *n* = 3 for (B–E and M), 6 for (F), 3–4 for (H and I), 5 for (J), 4 for (L). Scale bar, 50 µm for (B), 80 µm for (E), 100 µm for (M). Representative of two independent experiments.

To explore this innovative concept, after IT nanoSTING@Mn and LLL treatment for 20 days, tumor‐free mice were intranasally administered with nanoSTING@Mn either 14 days prior to (Group III) or on the same day (Group IV) as intravenous rechallenge with 1 × 10^5^ EL4 cells, as depicted in **Figure** **5**A. Naïve mice (Group I) and dual‐treated mice without intranasal nanoSTING@Mn (Group II) served as controls for tumor growth and systemic immunity, respectively. Lungs were collected two weeks after tumor rechallenge. Hematoxylin and eosin staining showed that alveolar structure, wall thickness, and microvasculature in Groups III and IV resembled those of healthy normal controls,^[^ ^62^ ^]^ whereas Groups I and II exhibited pathological thickening of alveolar walls (Figure 5B). Tumor burden, quantified using Image J (Figure 5C) and tumor nodule count (Figure S5a, Supporting Information) was significantly higher in Groups I and II than in Groups III and IV. Notably, even without intranasal nanoSTING@Mn, dual‐treated mice (Group II) showed a significant reduction in the lung metastases (*p* = 0.019), concomitant with significant increases in lung CD8^+^ T cells (Figure 5D,E), suggesting partial protection via systemic memory T‐cell responses. Strikingly, lung metastases were completely prevented in most of the mice receiving intranasal nanoSTING@Mn, especially when the adjuvant was administered two weeks prior to tumor rechallenge (Figure 5F). These observations argue strongly that directing CD8^+^ T cells to the lungs establishes robust local antitumor immunity, which can eliminate tumor “seeds” before their colonization. The greater efficacy observed in Group III over Group IV likely reflected the time required for early pulled CD8^+^ T cells to sufficiently differentiate into T_RM_ cells. Efficacy in Group IV might primarily relies only on effector T‐cell responses. In agreement with this, flow cytometry revealed significantly higher percentages of CD8^+^ T cells in Group III (38.0 ± 5.2%) and Group IV (32.9 ± 5.0%) than in Group I (15.4 ± 3.8%) and Group II (22.8 ± 2.6%) (Figure 5D). Representative flow cytometric gating and profiles are shown in Figure S4 (Supporting Information). Immunofluorescent staining of CD8^+^ T cells in the lung tissue corroborated these findings, showing the highest abundance of CD8^+^ T cells in Group III, followed by Group IV, then Group II, with the lowest in Group I (Figure 5E). Quantitatively, MFI of the immunofluorescent CD8^+^ T‐cell staining was significantly higher across all groups by intranasal nanoSTING@Mn administration, with 14.6‐fold increases in Group III (*p* < 0.0001) and 8.8‐fold in Group IV (*p* < 0.0001) in comparison to Group II (Figure 5E). Remarkably, intranasal nanoSTING@Mn gave rise to 100% survival at 36 days after intravenous EL4 tumor rechallenge, regardless of whether nanoSTING@Mn was administered two weeks prior to or concurrently with the rechallenge (Figure 5F). By contrast, all naïve mice succumbed within two weeks. The dual‐treated mice without intranasal nanoSTING@Mn (Group II) also exhibited significantly prolonged survival (*p* = 0.03) compared to naïve mice, highlighting a systemic memory antitumor immunity. To further evaluate long‐term immunity, Groups III and IV were rechallenged again with EL4 cells on day 48, and CD8⁺ T‐cell frequencies in the lungs were assessed at day 70. Flow cytometry revealed that CD8⁺ T cells were maintained at substantial levels in Group III (31.2 ± 1.5%) and Group IV (21.3 ± 1.0%) through day 70, indicating durable immune memory even after repeated tumor challenge (Figure S5b, Supporting Information). However, the finding that only intranasal nanoSTING@Mn‐treated mice achieved full protection marks another remarkable capacity of nanoSTING@Mn in deploying effector/memory CD8^+^ T cells to the lungs in an antigen‐independent fashion. This innovative strategy can be potentially extended to other tissues to preemptive metastasis control.

To determine essential roles for STING activation in lung epithelial cells in antigen‐independent pulmonary pulling of CD8^+^ T cells. The remarkable ability of nanoSTING@Mn to deploy T cells into the lungs in an antigen‐independent fashion propelled us to elucidate the underlying mechanism. We first compared the deployment capacity between nanoSTING@Mn and poly I:C, an agonist of intracellular TLR3 and a prominent intranasal adjuvant. As shown in Figure 5H,I, CD8^+^ T cells were significantly enriched in the mediastinal lymph nodes (mLNs) (1.7‐fold, *p* < 0.0001) and lungs (1.8‐fold, *p* < 0.0001) following intranasal nanoSTING@Mn compared to controls (Figure 5H,I). By contrast, intranasal poly I:C did not significantly increase T cells in either the mLNs or lungs as compared to controls, although slight upward trends were observed. In alignment with this, intranasal nanoSTING@Mn provoked the production of various chemokines and cytokines at levels substantially higher than poly I:C, for instance, *Ifn‐β1*, *Gm‐csf*, *Cxcl9*, *Ccl3*, and *Ccl2*, as demonstrated by q‐reverse transcriptase (RT)‐polymerase chain reaction (PCR) of lung tissues 16 h after intranasal delivery of the adjuvant (Figure 5J). This, in line with high levels of their cognate chemokine and cytokine receptor expression in CD8^+^ T cells in mice after the dual treatment, including *Ifnar1*, *Ifnar2*, *Csf2r*, *Cxcr3*, *Ccr3*, and *Ccr2* (Figure 5K), rationalizes their active migration and differentiation in the lungs following nanoSTING@Mn delivery.^[^ ^63^ ^]^ Based on these results and our previous investigation,^[^ ^33^ ^]^ we hypothesize that the ability of nanoSTING@Mn to activate the cGAS–STING pathway in lung epithelial cells is essential for pulling CD8^+^ T cells. Furthermore, bulk RNA sequencing analysis demonstrated that the expression profiles of apoptosis‐related genes, including *Bax*, *Fas*, *Casp3*, and *Casp8* after intranasal administration of nanoSTING@Mn were comparable to those of the control group. This indicates no evidence of apoptosis induction or toxicity in the respiratory epithelium following treatment. These findings further support the translational safety of the intranasal delivery approach. The results are provided in the Supporting Information.

To test this, on day 14 following the dual treatment, the mice were administered nanoSTING@Mn or poly I:C intranasally in the presence or absence of carbenoxolone (CBX), a cellular gap blocker^[^ ^33^ ^]^ (Figure 5G). The presence of CBX blocker significantly reduced the percentage of CD8^+^ T cells in both mLNs (1.4‐fold, *p* < 0.0001) and lungs (1.3‐fold, *p* < 0.05) compared to its absence, bringing about the levels almost down to the control levels (Figure 5H,I). By contrast, poly I:C administration with or without CBX induced essentially no change in CD8^+^ T‐cell infiltration in both mLNs and lungs (Figure 5H,I), as measured by flow cytometry gated as Figure S6a,b (Supporting Information). Apart from targeting different innate immune pathways, the difference between nanoSTING@Mn and poly I:C may be mainly ascribed to poly I:C's targeting antigen‐presenting cells (APCs) like alveolar macrophages and dendritic cells, whereas nanoSTING@Mn targets both APCs and lung epithelial cells. As we previously showed, pulmonary surfactant‐biomimetic liposomes used to encapsulate ADU‐S100–Mn complex interacted with surfactant protein A and D (SP‐A/D) and entered into alveolar macrophages via SP‐A/D‐mediated endocytosis to deliver the cargo, wherein Mn and ADU‐S100 were released from the endosomes, entered into cytoplasm, and fluxed into alveolar type II epithelial cells and then from epithelial cell type II to type I cells through cellular gap junctions owing to their small sizes, which is not accessible for the large molecules like poly I:C.^[^ ^33^ ^]^ CBX blocked the cellular gap junctions between alveolar epithelial cells and alveolar macrophages^[^ ^33^ ^]^ and thus abolished its activation of the STING pathway in lung epithelial cells. Because lung epithelial cells outnumber APCs by two orders of magnitude in noninflamed lungs, it is not surprising that the innate immune response induced by nanoSTING@Mn is much more robust than poly I:C, leading to high levels of various chemokines and cytokines.

Interestingly, these deployed T cells continuously differentiated into lung T_RM_ T cells as suggested by their increase three weeks post‐EL4 rechallenge (Figure 5G). Flow cytometric analysis revealed that lung T_RM_ cells characterized as CD3^+^CD8^+^CD69^+^CD103^+^ were elevated significantly following intranasal nanoSTING@Mn compared to either nanoSTING@Mn + CBX (2.0‐fold; *p* < 0.0001) or control groups (3.5‐fold; *p* < 0.0001) (Figure 5L). Representative flow cytometric gating and profiles are shown in Figure S6c (Supporting Information). We also stained lung T_RM_ cells by anti‐CD103 (red) and anti‐CD8 (orange) antibodies in lung tissue sections, which demonstrated maximum enrichment of lung T_RM_ cells following intranasal nanoSTING@Mn, manifested by a brighter, merged red‐orange color (Figure 5M). However, these T_RM_ cells diminished substantially after CBX treatment (Figure 5L,M), confirming their relevance to nanoSTING@Mn‐mediated pulling activity. Distinct from central and effector memory T cells, these lung T_RM_ cells are critical players in localized immune surveillance and eliminate the disseminated cancer cells before they can colonize. This proactive prevention strategy is highly clinically relevant, as lung T_RM_ cells in early stage human triple‐negative breast cancer are strongly correlated with improved patient prognosis.^[^ ^64^ ^]^ Collectively, these findings substantiate the potency of nanoSTING@Mn in mobilizing LLL‐revived CD8^+^ T cells to the lungs in an antigen‐independent fashion, presumably ascribed to its ability to activate the cGAS–STING pathway in lung epithelial cells. This paradigm‐shifting strategy can potentially redefine metastatic prevention by proactively and immunologically fortifying metastasis‐prone tissues through antigen‐independent deployment of T cells derived from primary tumor immunity.

## 3 Discussion

This study presents a dual therapeutic strategy that integrates LLL therapy with a novel biomimetic STING agonist nanoplatform (nanoSTING@Mn) to reprogram TIL metabolism and potentiate systemic antitumor immunity. Using a T‐cell lymphoma model, we demonstrate that nanoSTING@Mn robustly triggers infiltration of CD8⁺ T cells and monocytes into tumors, driving M1‐like macrophage polarization, suppressing Treg cells, and remodeling the immunosuppressive tumor microenvironment. Moreover, LLL, a noninvasive, drug‐free photo‐biomodulation, rewires mitochondrial metabolism in both CD8^+^ T cells and NK cells, expanding metabolically fit CD8⁺ T cells with progenitor‐exhausted and memory‐forming potential. However, other immune cell types such as macrophage, dendritic cells, neutrophils showed no changes in mitochondria after LLL treatment. Importantly, intranasal nanoSTING@Mn delivery enables antigen‐independent recruitment of these metabolically revived CD8⁺ T cells into the lungs, where they differentiate into T_RM_ T cells that provide durable, proactive protection against lung metastases. This convergence of biomaterial‐mediated innate immune activation with photonic metabolic modulation confers a powerful and versatile framework for reengineering antitumor immunity.

As an immunometabolic intervention, LLL offers distinct advantages over conventional pharmacologic approaches. Unlike agents such as PKM2 agonists that modulate cellular metabolism indirectly,^[^ ^14^ ^]^ LLL acts directly on mitochondrial respiratory complexes, enhancing ATP production and mitochondrial biogenesis even under hypoxic tumor microenvironments that typically impair immune function.^[^ ^17^, ^19^, ^20^, ^21^ ^]^ This localized, drug‐free modality minimizes systemic toxicity often associated with traditional immunotherapies such as ICBs, which can provoke severe immune‐related adverse events.^[^ ^2^ ^]^ Moreover, we have developed a wearable, stretchable LED device,^[^ ^65^ ^]^ enabling continuous or repeated light therapy at home. While the optimal duration of light exposure remains to be defined, the concept of safe, home‐based phototherapy holds strong translational appeal. Intriguingly, LLL selectively enhances the function of CD8^+^ T cells and NK cells, but not CD4^+^ T cells, mirroring recent studies of near‐infrared photo‐biomodulation preferentially improving CD8⁺ T‐cell activity, but not CD4^+^ T‐cell function ex vivo.^[^ ^24^ ^]^ This specificity may reflect the intrinsically higher metabolic demands of cytotoxic cells. It is plausible that LLL amplifies, but does not initiate, mitochondrial biogenesis, thus preferentially benefiting cell types already engaged in active energy remodeling. Our investigation demonstrates that LLL not only sustains CD8⁺ T‐cell functionality in the tumor, but also selectively enriches for the progenitor‐like and memory‐committed Tpex subset, critical for long‐term immune surveillance.^[^ ^53^ ^]^ This immunometabolic axis provides a novel therapeutic angle to overcome T‐cell exhaustion and dysfunction in solid tumors.

NanoSTING@Mn exerts its immune‐stimulatory effects by coactivating cGAS with Mn^2^⁺ and STING with its agonist ADU‐S100.^[^ ^10^ ^]^ Unlike TLRs, which are largely restricted to immune cells,^[^ ^66^ ^]^ the cGAS–STING pathway is expressed in nearly all nucleated cells, including epithelial and tumor cells.^[^ ^33^ ^]^ In noninflamed tissues or immune‐desert tumors, this widespread expression makes the cGAS–STING pathway a more effective target for initiating localized innate immune responses. This may be the reason behind the superior ability of intranasal nanoSTING@Mn to recruit immune cells to the lungs as compared to poly I:C. To the best of our knowledge, nanoSTING@Mn is the first mucosal adjuvant capable of antigen‐independent T‐cell deployment to the lungs, a strategy previously thought unachievable without known antigen specificity.^[^ ^61^ ^]^ T‐cell deployment to metastatic‐prone organs, such as the lungs, can preemptively eliminate disseminated tumor cells upon arrival, representing a powerful strategy to prevent metastasis.

In summary, this study establishes a transformative platform that combines photonic metabolic reprogramming with cGAS–STING‐driven innate immune activation to overcome two key barriers in cancer immunotherapy: T‐cell exhaustion and insufficient systemic immune coverage. By sustaining T‐cell fitness, promoting memory formation, and enabling antigen‐independent targeting of at‐risk tissues, the platform opens a new frontier for both treatment and prevention of metastatic disease. Importantly, no clinical signs of autoimmunity, such as weight loss, were observed in treated mice despite prolonged T‐cell activation. Nonetheless, comprehensive evaluation of long‐term safety in extended studies will be essential to fully validate the translational potential of this approach. Future studies should explore this approach to other tumor types, mucosal sites, and adjuvant combinations to further enhance its therapeutic potential. Although our current focus was on the intravenous rechallenge model, we do agree that testing in more physiologically relevant models such as orthotopic or spontaneous metastases model would provide insight into the therapeutical efficacy of intranasal administration of nanoSTING@Mn.

## 4 Experimental Section

### Cells and Animals

EL4 cells were obtained from the American Type Culture Collection and maintained in Dulbecco's Modified Eagle's Medium (DMEM) supplemented with 10% fetal bovine serum (FBS), 100 µg mL^−1^ penicillin, and 100 µg mL^−1^ streptomycin. All cells were cultured at 37 °C in a humidified incubator with 5% CO_2_. C57BL/6 mice at age of 6–8 weeks were purchased from Charles River Laboratories. The animal studies were approved by the Massachusetts General Hospital (MGH) Institutional Animal Care and Use Committee (2008N000173).

### Nanoparticle Uptake Study

EL4 cells were plated at a density of 5 × 10^5^ cells mL^−1^ in a 29 mm glass bottom dish and treated with free SRB (Sigma‐Aldrich) or nanoSRB for 6 and 24 h at the equivalent concentration of SRB (1 µg mL^−1^). The cells were washed thrice with phosphate‐buffered saline (PBS) and imaged using confocal microscopy (*n* = 3) (Olympus FV1000, UPLSAPO 60XW). NanoSRB uptake was also evaluated in C57BL6 mice bearing EL4 tumor. The tumor cells were subcutaneously inoculated into the right back flank with 1 × 10^6^ per 100 µL of EL4 cells. Once the tumor reached 200 mm^3^, SRB and nanoSRB at an equivalent dose of 20 µg SRB per tumor were IT injected. At the defined time interval, tumors were excised and imaged using confocal microscopy (*n* = 4).

### Bone‐Marrow‐Derived Macrophage (BMDM) Differentiation and Coculture with CD4^+^ T Cells

Bone marrow cells were harvested from the mouse hind leg bones and cultured at 5 × 10^5^ cells mL^−1^ in DMEM medium supplemented with 10% FBS and 20 ng mL^−1^ macrophage colony‐stimulating factor in 24‐well plates. After 3 days, most of the cells were differentiated into monocytes and these cells were further activated with OVA (100 µg mL^−1^) either alone or together with nanoSTING@Mn (5 µg mL^−1^) for 72 h, followed by staining with *Ly6c*, *CD86*, and *Cellrox* to determine M1 macrophage phenotype. Additionally, naïve CD4^+^ T cells were isolated from mouse spleens using the MagniSort Negative Selection Kit (Invitrogen), following the manufacturer's protocol. The purified naïve T cells were activated with anti‐CD3/anti‐CD28‐conjugated Dynabeads magnetic beads (Gibco) at a bead‐to‐cell ratio of 2:1 for 3 days in 10% FBS complete DMEM medium. To evaluate Foxp3 suppression by activated BMDM, the aforementioned activated CD4⁺ T cells were cocultured with nanoSTING@Mn‐activated BMDMs in the presence of OVA at a 1:4 ratio. Intracellular Foxp3 expression was assessed on day 6 after incubation with activated BMDM. These T cells were first marked by surface staining with anti‐CD4 and anti‐CD25 antibodies and then fixed, followed by intracellular staining for Foxp3 according to the manufacturer's instructions before flow cytometry (*n* = 3). The following antibodies used in the experiment were purchased from BioLegend: APC/Cyanine 7 F4/80 (catalog no: 123117), Brilliant Violet 510 CD11c (catalog no: 117337), Brilliant Violet 421 CD86 (catalog no: 105031), PE *Ly6c* (catalog no: 128007), Alexa Fluor 488 Foxp3 (catalog no: 126405), APC/Cyanine 7 CD4 (catalog no: 100413), APC CD25 (catalog no: 113708). Reagents for intracellular Foxp3 staining were obtained from BioLegend, and staining was performed according to the manufacturer's instructions. Cellrox (Invitrogen, catalog no: C10444) was stained for 30 min at 37 °C. All other antibodies were stained for 30 min on ice.

### Assessment of Antitumor Immunity

C57BL/6 mice were inoculated subcutaneously with 1 × 10^6^ per 100 µL of EL4 cells in the right back flank. Once the tumor reached 100 mm^3^, mice were randomly divided into 5 groups and treated with control (saline), STING agonist ADU‐S100, nanoSTING, STING@Mn, or nanoSTING@Mn. For nanoSTING@Mn preparation, ADU‐S100 was complexed with MnCl_2_·4H_2_O in methanol and centrifuged at 18 000 *g* for 20 min as previously described.^[^ ^10^ ^]^ The ADU–Mn pellet was resuspended in citrate buffer (pH 4.0). A lipid film was prepared by dissolving 1,2‐dipalmitoyl‐*sn*‐glycero‐3‐phosphocholine, 1,2‐dipalmitoyl‐snglycero‐3‐phospho‐(1′‐rac‐glycerol), 1,2‐dipalmitoyl‐*sn*‐glycero‐3‐phosphoethanolamine‐*N*‐[methoxy(polyethyleneglycol)‐2000], and cholesterol (10:1:1:1 molar ratio) in chloroform, followed by rotary evaporation at 37 °C to form a thin film.^[^ ^33^, ^62^ ^]^ The film was hydrated with the ADU–Mn complex for 40 min at 45 °C and sonicated for 40 min at room temperature. NanoSTING@Mn was collected using a 100 kDa MWCO centrifugal filter at 2000 *g* for 5 min.

The equivalent dose of STING agonist was 5 µg per mouse, and the treatments were given through the IT injection at days 0 and 3. Body weight and tumor volume were monitored every 2 days until day 8 after the first treatment. Tumor sizes were measured using a vernier caliper to measure the length and width of the tumor, and tumor volume was calculated using the equation: volume = length × width^2^ × 0.5 (*n* = 5). Tumors were excised on day 8 and subject to cytokine analysis using qRT‐PCR or stained with CD3 and CD8 antibodies to analyze CD8^+^ T cells by flow cytometry (*n* = 3).

To investigate the synergistic antitumor effects of nanoSTING@Mn combined with LLL, mice bearing tumors (≈100 mm^3^) were randomly assigned to one of the five groups: 1) control (saline), 2) IT nanoSTING@Mn, 3) IT nanoSTING@Mn with LLL applied to dLNs, 4) IT nanoSTING@Mn with LLL applied to the tumor (Tumor), and 5) IT nanoSTING@Mn with LLL applied to both tumor and dLNs (dLNs + Tumor). Tumor cells were inoculated, and nanoSTING@Mn was administered as above. LLL was applied using an 810 nm near‐infrared laser (PhotoThera, Inc.) calibrated to deliver a fluence of 3 J cm^−^^2^ per session, with each session lasting 2 min. Hair over the treatment areas (tumor and/or inguinal dLNs) was removed with depilatory cream 24 h before the first irradiation to ensure efficient light penetration. LLL was administered externally on days 0, 4, 8, and 12. Body weight and tumor volume were recorded every 2 days as above (*n* = 10). Tumors were excised on day 12 or 20 and prepared as single‐cell suspensions. The cells were stained with CD3, CD8 antibodies, and MitoTracker Red to assess CD8⁺ T‐cell frequency and mitochondrial activity by flow cytometry. For NK cells, the cells were stained with CD3 and NK1.1 antibodies, followed by flow cytometry (*n* = 3). Frozen tumor sections were incubated with CD8α antibody and DAPI to visualize CD8^+^ T cells using confocal microscopy (*n* = 3).

### Intranasal NanoSTING@Mn to Deploy T Cells to the Lungs in Preventing Lung Metastases

Mice that had treated the primary tumor 20 days after treatment with nanoSTING@Mn + LLL (dLNs + Tumor) were intravenously rechallenged with EL4 tumor cells at 1 × 10^5^ cells per 100 µL on day 34. Several experimental conditions were evaluated: mice received intranasal nanoSTING@Mn (15 µg of STING agonist per mouse) either two weeks prior to rechallenge (Group III) or on the day of rechallenge (Group IV). Control groups included naive mice (Group I) and tumor‐free mice that were rechallenged without intranasal nanoSTING@Mn (Group II). Two weeks after EL4 cell rechallenge, lungs were collected and histologically examined for metastases (*n* = 3). To further evaluate long‐term immunity, Groups III and IV mice were further rechallenged with EL4 cells on day 48, and CD8⁺ T‐cell frequencies in the lungs were assessed at day 70 (*n* = 3). Lung single‐cell suspensions were prepared and stained with CD3 and CD8 antibodies for flow cytometric analysis (*n* = 3). Frozen lung sections were incubated with CD8α antibody and DAPI for confocal imaging of CD8^+^ T cells (*n* = 3). Some mice were monitored for survival until day 70 (*n* = 6). In a separate experiment, mice bearing tumors were treated with IT nanoSTING@Mn and LLL (dLNs + Tumor) and intranasally administered with nanoSTING@Mn or poly I:C on day 14 in the presence or absence of CBX. CBX (400 µg per mouse), a blocker of the cellular gap junctions, was administered intraperitoneally for 4 consecutive days starting on day 12 post‐tumor‐inoculation. Lungs and mLNs were excised on day 21 and processed into single‐cell suspensions, and stained with CD3 and CD8 antibodies to assess CD8⁺ T‐cell frequency by flow cytometry (*n* = 3–4).

To assess lung T_RM_ CD8^+^ cells following intranasal nanoSTING@Mn, some mice received an intravenous rechallenge with EL4 cells on day 14, three weeks after which the lungs were excised, and lung T_RM_ cells were analyzed by immunofluorescence or flow cytometry. Lung T_RM_ CD8^+^ T cells were measured after excluding vascular CD8^+^ T cells per a published protocol.^[^ ^67^ ^]^ CD3^+^CD8^+^CD69^+^CD103^+^ cells were defined as lung T_RM_ CD8^+^ T cells by flow cytometry (*n* = 4).

### Cytokine and Chemokine Measurements

Tumor tissues were cut into small pieces 24 h after the second dose of IT administration of the indicated STING agonists. Total RNA was extracted using Aurum Total RNA Mini Kit (Bio‐Rad) and reverse‐transcribed (Life Technologies), followed by amplification with real‐time qPCR using a SYBR green PCR kit (Roche) according to the manufacturer's instructions. Glyceraldehyde 3‐phosphate dehydrogenase was used as an internal control during the experiment. To measure cytokines and chemokines induced by adjuvants in the lungs, C57BL/6 mice were intranasally administered with 15 µg of nanoSTING@Mn or poly I:C (*n* = 5). After 16 h, flash‐frozen lung tissues were homogenized and RNA was extracted and reverse‐transcribed, followed by real‐time qPCR as above. Primers used are listed in Table S1 (Supporting Information).

### Single‐Cell Suspensions Prepared from Tissues for Flow Cytometry

Lungs, mLNs, and tumor tissue were collected on the indicated days and cut into small pieces. The lung and tumor tissues were treated with DNAse I (0.1 mg mL^−1^) and collagenase D (1 mg mL^−1^) at 37 °C for 1 h. The resulting cell suspension was treated with ACK lysis buffer for RBC lysis and filtered through a 40 µm strainer to obtain single‐cell suspensions. The mLNs were mechanically dissociated, and single‐cell suspensions were prepared by directly passing through a 40 µm strainer. The following anti‐mouse antibodies were used for flow cytometry and purchased from BioLegend unless otherwise specified: fluorescein isothiocyanate (FITC) CD3 (Clone: 17A2; catalog no: 100204), PE CD3 (clone: 17A2; catalog no: 100206), Pacific Blue CD8a (clone: 53‐6.7; catalog no: 100725), PE NK‐1.1 (clone: S17016D; catalog no: 156504), FITC CD8b (clone: YTS156.7.7; catalog no: 126606), Brilliant Violet 510 CD103 (clone: 2E7; catalog no: 121423), APC/Cyanine7 CD69 (clone: H1.2F3; catalog no: 104526), APC CD8a (clone: 53‐6.7; catalog no: 100712), and PE/Cyanine7 CD3 (clone: 17A2; catalog no: 100219). MitoTracker Red CMXRos Dye (catalog no: M46752) was purchased from Invitrogen. Stained cells were acquired on a FACSAria II (BD) and analyzed using FlowJo software (Tree Star).

### ScRNA Sequencing and Data Analysis

Tumors were excised on day 12 for scRNA sequencing. Single‐cell suspensions were prepared using the previously described method.^[^ ^10^ ^]^ For analysis, tumors from three treated mice in each group were collected, and their TILs were pooled. TILs were isolated through the density‐based Ficoll‐Paque gradient and suspended in PBS. Propidium iodide staining was used to exclude dead cells, and at least 10 000 viable cells per sample were submitted for scRNA sequencing by the MGH NextGen Sequencing core. ScRNA‐seq library was prepared using the Chromium Single Cell 3′ Reagent Kits v3.0 (10x Genomics) on the Chromium Controller, according to the manufacturer's protocol. Sequencing was conducted on the Illumina NextSeq 2000 platform. Raw sequencing data were processed using the Cell Ranger software suite (10x Genomics) for alignment, deduplication, and identification of cut sites for each sample. The resulting data from individual samples were integrated in R (RStudio) using the Seurat package. Batch effects were corrected using the Harmony algorithm. Cell type annotation was performed using the SingleR and CellRex packages, based on reference transcriptomic datasets. Cell types were computed by principal component analysis, UMAP dimension reduction, and visualized by Loupe Browser v7.0. Differentially expressed genes were calculated using the Seurat “FindAllMarkers” function to test genes with >0.3‐fold difference (log‐scale). The DEGs between CD8 subsets were clustered to investigate GOBP gene sets using GSEA with the tidyverse, clusterProfiler, and GSEABase packages. Single‐cell type and pseudotime trajectory plot were constructed with Monocle2. Heatmaps and dotplots were performed using the ComplexHeatmap package or Seurat Dotplot function. Genes related to OXPHOS, T‐cell memory, and mitochondrial activity were pulled from gene sets of GO terms, normalized by sample using average expression and visualized by the ComplexHeatmap package.

### Immunofluorescent Staining and Confocal Microscopy

Tumor and lung tissues were embedded in an optimal cutting temperature compound (Sakura Finetek) and sectioned into 5 µm frozen slices. The sections were blocked with a blocking solution and incubated with APC anti‐mouse CD8α antibody to label CD8^+^ T cells (Biolegend, clone: 53‐6.7, catalog no: 100712) for 2 h at 4 °C. For Foxp3 staining in tumor tissue, sections were permeabilized with 0.1% Triton X‐100 for 15 min at room temperature, blocked with a blocking buffer, and stained with Alexa Fluor 488 anti‐mouse/rat/human Foxp3 antibody (Biolegend, clone: 150D, catalog no: 320012) for 2 h. To visualize lung T_RM_ cells, slides were blocked with a blocking buffer and stained overnight with CD8 rat monoclonal antibody (Invitrogen, catalog no: MA1‐10301) and CD103 rabbit polyclonal antibody (Invitrogen, Clone: 53‐6.7, catalog no: PA5‐99400). Subsequently, sections were stained with Alexa Fluor 555 goat anti‐rat IgG (Invitrogen, catalog no: A21434) and Alexa Fluor 647 goat anti‐rabbit IgG (Invitrogen, catalog no: A21245) for 1 h at room temperature. All slides were washed 3 times with PBS, mounted with ProLong Antifade Mountant containing DAPI (Life Technologies), and imaged by confocal microscopy.

### Histology

Lungs were collected on day 48 post‐tumor‐cell inoculation subcutaneously, fixed in 10% v/v formalin for 24 h, transferred into 70% ethanol, and stained with hematoxylin and eosin following a standard procedure. Slides were scanned and analyzed using NanoZoomer (Hamamatsu).

### Statistical Analysis

The results were expressed as means ± standard error of the mean (SEM). One‐ or two‐way analysis of variance (ANOVA) analysis, followed by Bonferroni's multiple comparisons test, was used to evaluate differences among groups. Experiments were repeated at least once or as indicated in the figure captions. Statistical analysis was carried out using GraphPad Prism 9.0 (GraphPad Software).

## Conflict of Interest

The authors declare no conflict of interest.

## Author Contributions

Conceptualization and supervision, M.X.W.; investigation and data analysis, A.B., S.G., Z.Z., Y.D., P.U., Q.Z., Y.L., Z.‐T.L., Z.W., and M.X.W.; bioinformatic analysis, S.G.; and writing, A.B. and M.X.W. M.X.W. secured funding for the study.

## Supporting information



Supporting Information

## Data Availability

The data that support the findings of this study are available from the corresponding author upon reasonable request.
