# Supplementary material for: Light Metabolically Reprograms CD8+ T Cells to Potentiate STING‐Driven Tumor Eradication and Prevent Metastasis
Source: Adv Sci (Weinh). 2025 Oct 23;13(1):e15121. doi: 10.1002/advs.202515121 (PMC12767170; doi:10.1002/advs.202515121)
Supplement: Supplementary file 1 — Supporting Information [file ADVS-13-e15121-s001.docx]

**Additional method and result**

**Evaluation on antitumor effect of LLL alone on EL4 tumor model.**

To evaluate the antitumor effect of LLL, we applied 810 nm of LLL directly and non-invasively at a power density of 3 J/cm² to the Tumor every day for a period of 6 days. Our result demonstrated that LLL by itself did not show antitumor effect and tumor volume was comparable to untreated controls, suggesting that LLL does not exert a direct antitumor effect (Fig. S2a).

**RNA sequencing of lung epithelial cells**

To evaluate the safety of lung epithelial cells following intranasal administration of NanoSTING@Mn, lung tissues were collected and processed into single-cell suspensions. Epithelial cells were enriched by flow cytometry–based sorting using the markers CD45⁻EpCAM⁺. The purified epithelial cell population was then subjected to bulk RNA sequencing to assess apoptosis marker following treatment.


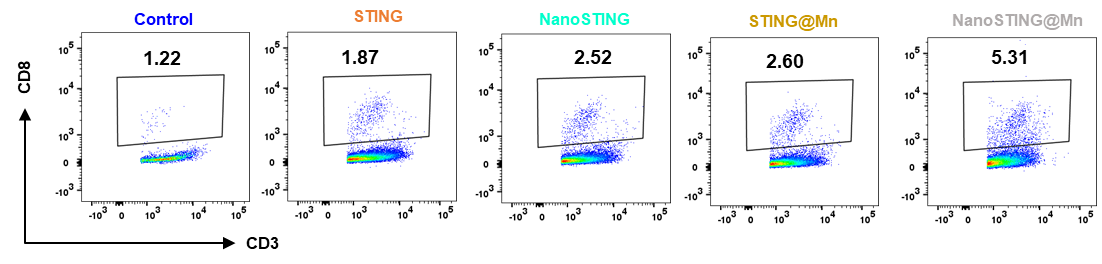


**Supplementary Figure S1.** Representative scatter plots of CD8 distribution in tumor microenvironment after intratumoral injection of NanoSTING@Mn related to **Fig 1E**.


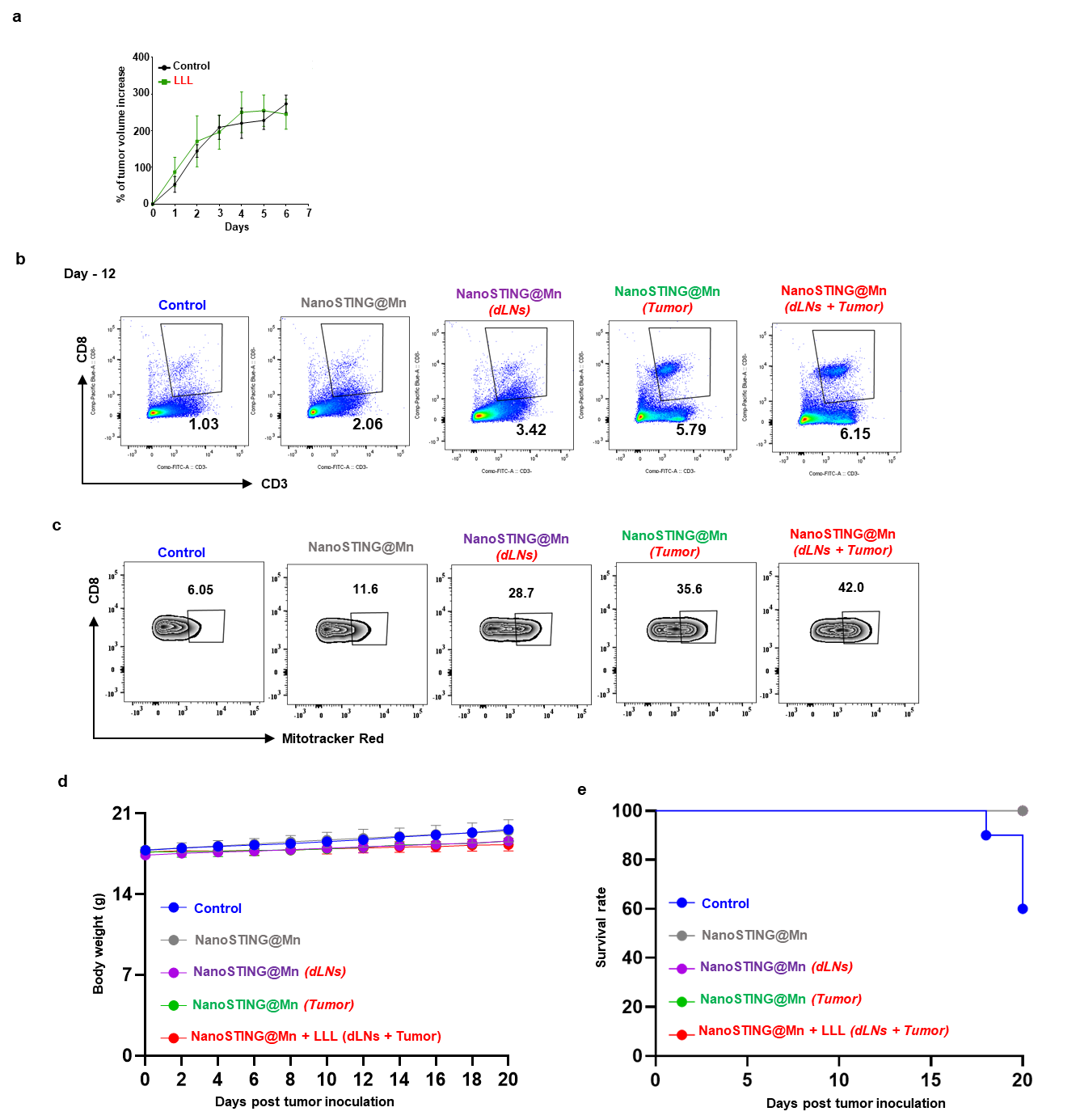


**Supplementary Figure S2. (a)** EL4 cells were inoculated and grew into tumors. Tumor-bearing C57BL/6 mice were treated with LLL every day until day 6, and tumor volume was monitored over time. **(b)** Representative scatter plots of CD8 distribution in tumor microenvironment after combined effect of LLL and NanoSTING@Mn and LLL related to **Fig 2C. (c)** Representative scatter plots of Mitotracker Red staining on gated CD8 T cells distribution in tumor microenvironment after combined effect of LLL and NanoSTING@Mn and LLL related to **Fig 2D**. Body weight **(d)** evaluation on every two days following treatment with distinct groups until day 20. **(e)** Kaplan–Meier survival curves of the treatment groups up to 20 days. Note, the survival curves are overlapped for all treated groups.


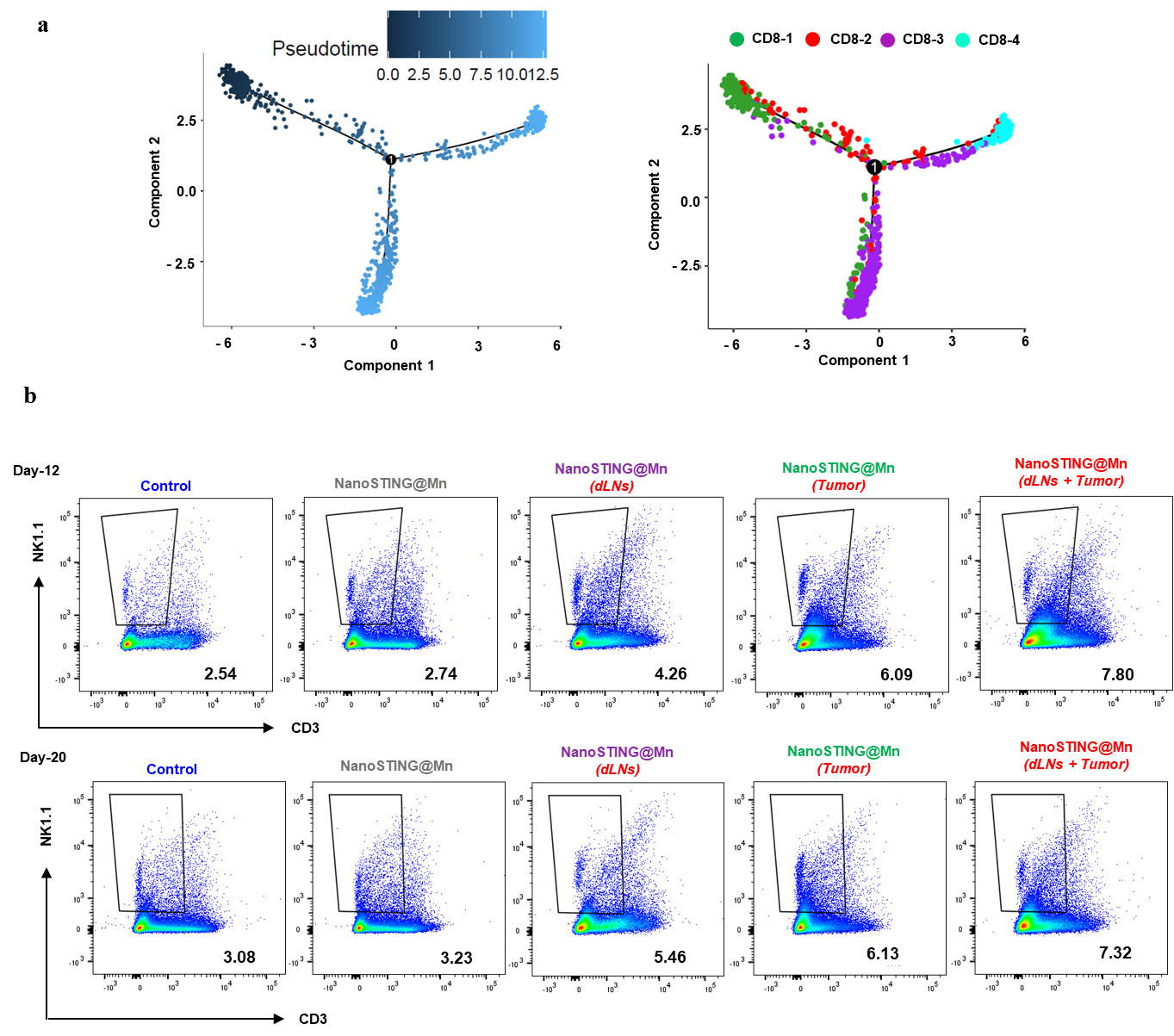


**Supplementary Figure S3. (a)** Pseudotime trajectory plot analysis demonstrating the differentiation progress of CD8 T cells after treatment. **(b)** Representative scatter plots of NK1.1 cells distribution in tumor microenvironment after administration of NanoSTING@Mn and LLL related to **Fig 4D**.


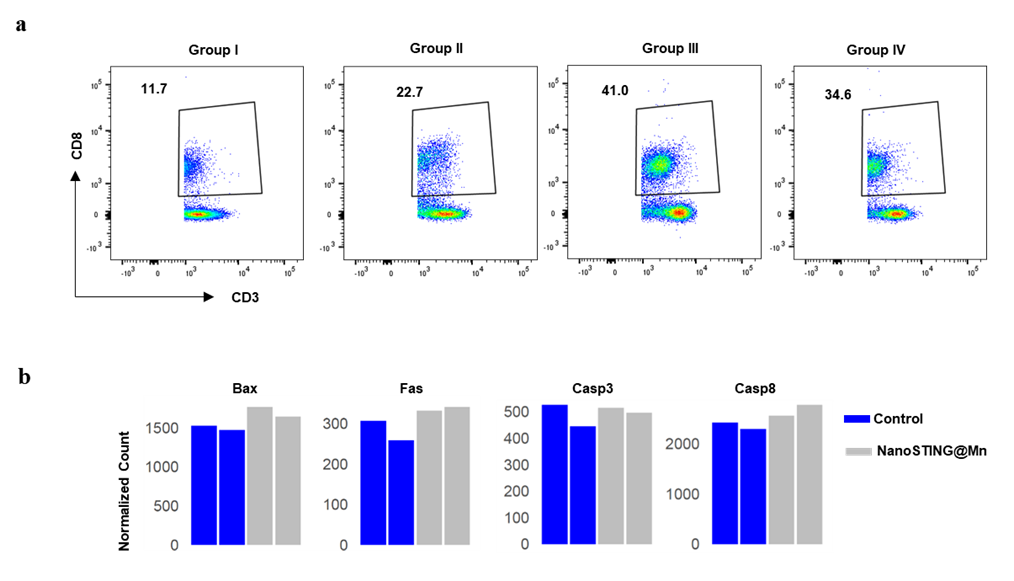


**Supplementary Figure S4. (a)** Representative scatter plots of CD8 T cells distribution in lungs after intranasal administration of NanoSTING@Mn related to **Fig 5D. (b)** Apoptosis marker in lung epithelial tissue after intranasal administration of NanoSTING@Mn.


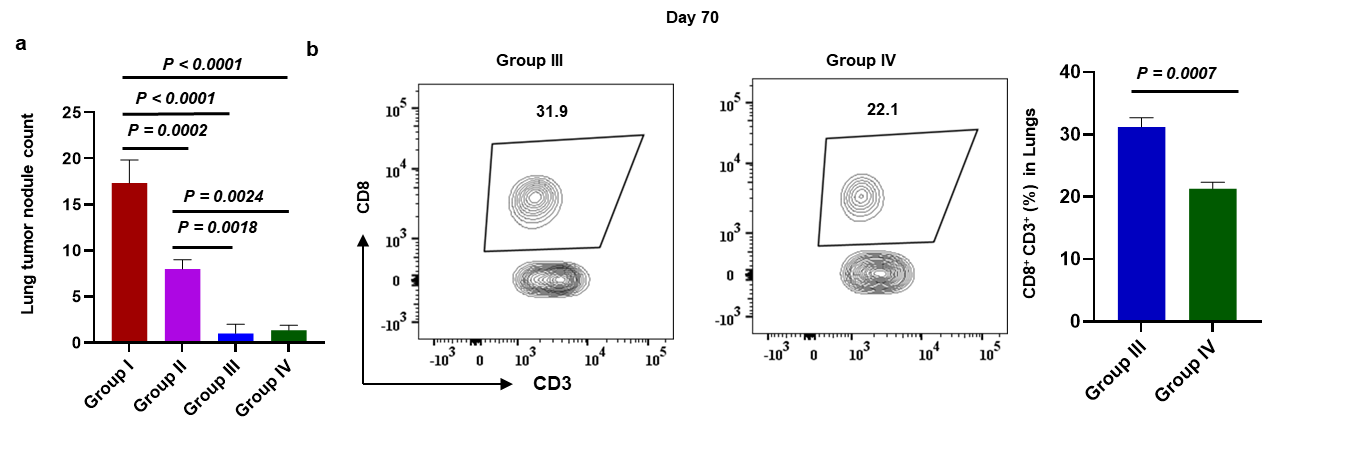


**Supplementary Figure S5. (a)** Lung tumor nodule count related to **fig. 5C**. **(b)** Flow cytometry analysis of CD8^+^CD3^+^ T cells (%) in lung tissue at day 70 related to **fig. 5F**.


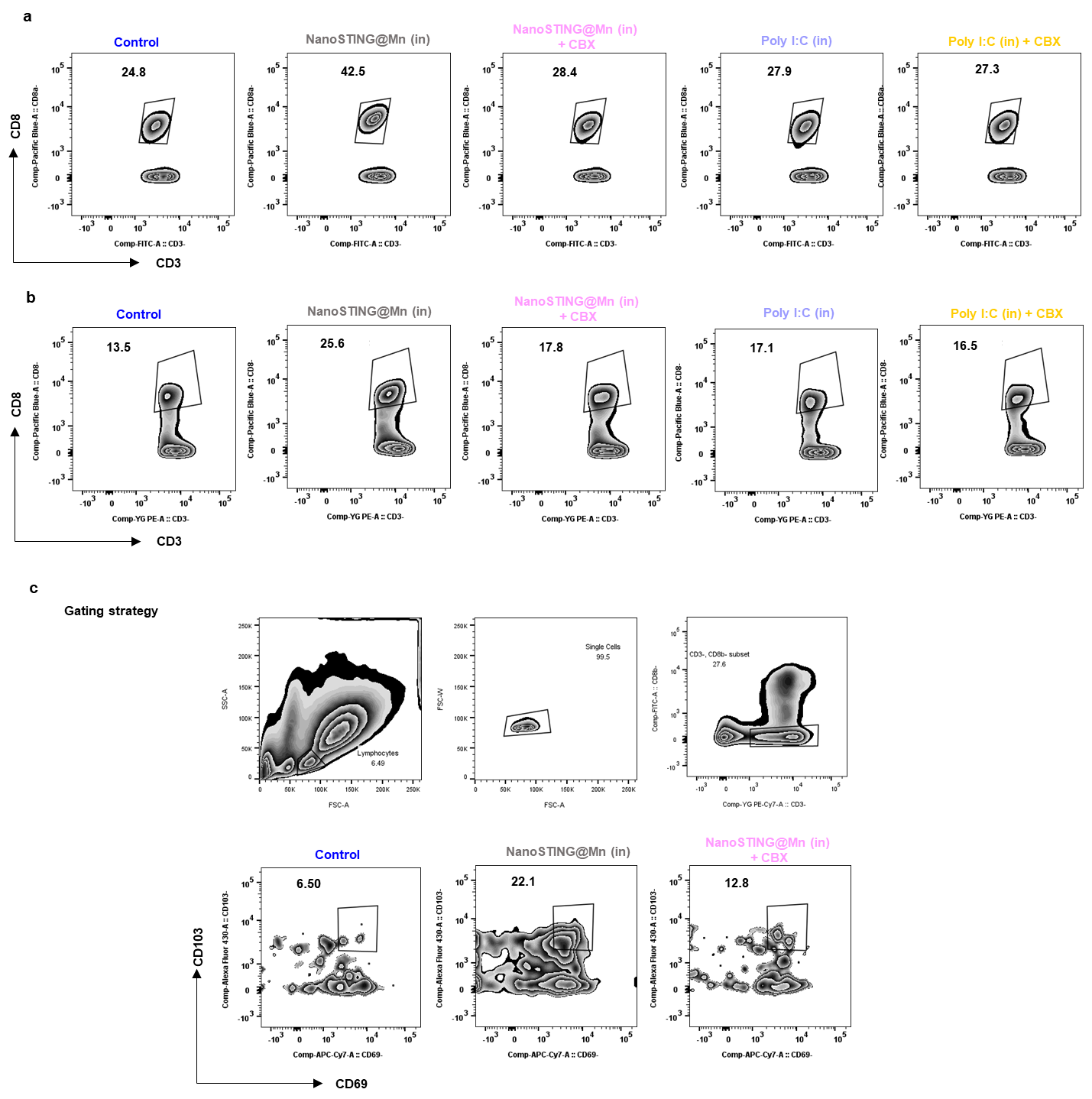


**Supplementary Figure S6.** Representative scatter plots showing the distribution of CD8 T cells in MLNs **(a)** and lungs **(b)** following intranasal administration of NanoSTING@Mn and Poly I:C with or without CBX, corresponding to **Fig. 5H** and **I**, respectively. **(c)** The gating strategy for lung T_RM_ cells is illustrated, with representative scatter plots displaying lung T_RM_ following concurrent intranasal administration of NanoSTING@Mn with or without CBX and subsequent intravenous challenge with EL4 cells, corresponding to **Fig. 5J**.

**Table S1.** Primer sequences for qPCR

| Gene |  | Sequence |
| --- | --- | --- |
| *Gapdh* | F | ATCAAGAAGGTGGTGAAGCA |
|  | R | AGACAACCTGGTCCTCAGTGT |
| *Ifnβ1* | F | AGCTCCAAGAAAGGACGAACA |
|  | R | GCCCTGTAGGTGAGGTTGAT |
| *Gmcsf* | F | GAAGCATGTAGAGGCCATCA |
|  | R | GAATATCTTCAGGCGGGTCT |
| *TNF-α* | F | CCTGTAGCCCACGTCGTAG |
|  | R | GGGAGTAGACAAGGTACAACCC |
| *Cxcl-9* | F | TCCTTTTGGGCATCATCTTCC |
|  | R | TTTGTAGTGGATCGTGCCTCG |
| *Cxcl-10* | F | CCAAGTGCTGCCGTCATTTTC |
|  | R | TCCCTATGGCCCTCATTCTCA |
| *Ccl2* | F | TCTGGGCCTGCTGTTCACA |
|  | R | CCTACTCATTGGGATCATCTTGCT |
| *Ccl3* | F | TGTACCATGACACTCTGCAAC |
|  | R | CAACGATGAATTGGCGTGGAA |
